# Influenza and Pneumococcal Vaccination Uptake in Patients with Rheumatoid Arthritis Treated with Immunosuppressive Therapy in the UK: A Retrospective Cohort Study Using Data from the Clinical Practice Research Datalink

**DOI:** 10.1371/journal.pone.0153848

**Published:** 2016-04-20

**Authors:** Ruth Costello, Kevin L. Winthrop, Stephen R. Pye, Benjamin Brown, William G. Dixon

**Affiliations:** 1 Arthritis Research UK Centre for Epidemiology, Centre for Musculoskeletal Research, Institute for Inflammation and Repair, Manchester Academic Health Science Centre, The University of Manchester, Manchester, United Kingdom; 2 Division of Infectious Diseases, Oregon Health and Science University, Portland, Oregon, United States of America; 3 Health eResearch Centre, Manchester Academic Health Science Centre, The University of Manchester, Manchester, United Kingdom; VU University Medical Center, NETHERLANDS

## Abstract

**Introduction:**

Guidelines for the management of rheumatoid arthritis (RA) recommend using influenza and pneumococcal vaccinations to mitigate infection risk. The level of adherence to these guidelines is not well known in the UK. The aims of this study were to describe the uptake of influenza and pneumococcal vaccinations in patients with RA in the UK, to compare the characteristics of those vaccinated to those not vaccinated and to compare vaccination rates across regions of the UK.

**Methods:**

A retrospective cohort study of adults diagnosed with incident RA and treated with non-biologic immunosuppressive therapy, using data from a large primary care database. For the influenza vaccination, patients were considered unvaccinated on 1st September each year and upon vaccination their status changed to vaccinated. For pneumococcal vaccination, patients were considered vaccinated after their first vaccination until the end of follow-up. Patients were stratified by age 65 at the start of follow-up, given differences in vaccination guidelines for the general population.

**Results:**

Overall (N = 15,724), 80% patients received at least one influenza vaccination, and 50% patients received a pneumococcal vaccination, during follow-up (mean 5.3 years). Of those aged below 65 years (N = 9,969), 73% patients had received at least one influenza vaccination, and 43% patients received at least one pneumococcal vaccination. Of those aged over 65 years (N = 5,755), 91% patients received at least one influenza vaccination, and 61% patients had received at least one pneumococcal vaccination. Those vaccinated were older, had more comorbidity and visited the GP more often. Regional differences in vaccination rates were seen with the highest rates in Northern Ireland, and the lowest rates in London.

**Conclusions:**

One in five patients received no influenza vaccinations and one in two patients received no pneumonia vaccine over five years of follow-up. There remains significant scope to improve uptake of vaccinations in patients with RA.

## Introduction

Patients with rheumatoid arthritis (RA) are known to have a two-fold increased risk of infections compared to the general population [1]. This is thought to be due to the disease itself, shared risk factors such as smoking, comorbidities and immunosuppressive treatment [2]. For certain infections such as influenza and pneumonia, vaccinations may confer protection [3, 4]. Studies have shown that influenza and pneumococcal vaccinations are safe in patients with RA and produce an antibody response despite immunosuppressive medication [5].

The European League Against Rheumatism (EULAR) recommendations for vaccination in patients with rheumatic diseases recommend vaccination during stable disease, and ideally prior to starting disease-modifying anti-rheumatic drug (DMARD) therapy. A pneumococcal vaccination and annual influenza vaccinations are recommended for patients with RA being treated with immunosuppressive medication. It is unknown whether patients should have booster pneumococcal vaccinations [5]. The UK guidelines recommend a single vaccination for immunocompromised patients [6] and the US guidelines recommend revaccination 5 years after first vaccinations for those below 65 years of age and at age 65 years, or later if at least 5 years have elapsed since the previous dose [7].

Previous studies have investigated the uptake of both influenza and pneumococcal vaccines in patients with rheumatic diseases [8–20]. These have typically been small single centre studies mostly conducted in the US and Europe, often reliant on self-report of vaccinations. They have shown suboptimal uptake of vaccinations, especially pneumococcal vaccinations [9–13, 18–20]. At a national level in the UK, the Department of Health publishes data on vaccination uptake [21], but the figures are not broken down by indication. Only one study in the US has looked at whether patients with rheumatic diseases are being vaccinated prior to starting immunosuppressive therapy [15].

Patients with RA are a high risk group with specific guidelines about vaccination and infection prevention, with little data about vaccination uptake at a national level in the UK. Given this, the aims of this study were to describe the influenza and pneumococcal vaccination uptake in patients with incident RA in the UK, to compare the characteristics of those vaccinated to those who were not vaccinated and to compare vaccination coverage across regions of the UK.

## Materials and Methods

This study used data from the Clinical Practice Research Datalink (CPRD)—a large database of anonymised primary care electronic medical records from general practitioners in the UK. As of March 2011, it contains data for over 12 million patients from the mid 1980’s onwards [22]. The records provide a rich source of information on clinical diagnoses and symptoms, immunisations, prescriptions, referrals and tests. The CPRD use data quality metrics to ensure the quality of the data at the individual level, by indicating poor data recording or non-continuous follow-up with an acceptability flag, and at the practice level, by indicating when a practice’s data is up to research standard.

### Definition of patients with incident RA

This was a retrospective cohort study, with the study period 1^st^ January 2000 to 31^st^ December 2013. To be included in the cohort patients needed to be i) diagnosed with RA for the first time within the study window, identified using Read codes (see S1 Table for codelist) according to a validated definition [23], ii) treated with immunosuppressive therapy at some point during follow-up (identified through product codes (medication codes) for methotrexate, sulfasalazine, leflunomide, hydroxychloroquine and other non-biologic DMARDs) (see S2 Table for codelist). iii) aged 18 years or over and iv) have at least 12 months electronic medical record data prior to entry to the study, to allow determination of baseline characteristics. To ensure data quality, data from patients deemed unacceptable and data prior to the practice being up to standard was not used. Patients entered the study on the date of their RA diagnosis, defined as the first Read code for RA. Follow-up time was censored at either death, transfer out of the practice, when the GP practice stopped contributing CPRD data, or the time of data extraction from CPRD, whichever came sooner.

### Vaccination status

Influenza vaccine is an annual vaccine that is adjusted each year depending on the strains of influenza predicted for the influenza season, estimated to be 1st September to 31st March. Exposure to influenza vaccination was therefore time dependent. On 1st September all individuals had an unvaccinated status and, upon vaccination their status changed to vaccinated. On 31st August their status returned to unvaccinated and this was repeated each year. Vaccination for influenza was identified through Read and product codes (see S3 Table for codelists). If there was more than one entry for immunisation during the season the first date was used. Pneumococcal vaccination (PPV23) was identified using Read and product codes (see S4 Table for codelists). Following pneumococcal vaccination patients were considered vaccinated for the rest of follow-up during the study time-period. Any repeat vaccinations remained in the database but did not alter vaccination status. For both vaccines, patients were classified by whether they had any vaccinations during follow-up and the number of vaccinations during follow-up. The expected number of vaccinations during follow-up was calculated for influenza vaccinations.

### Covariates

Age at baseline (date of RA diagnosis) was calculated by subtracting year of RA diagnosis from year of birth. Patients were then divided into those above and below 65 years of age, due to differences in vaccination guidelines in the general population. Baseline covariates were determined using data from at least 12 months prior to cohort entry. Baseline smoking status (ever smoker vs never smoker) was identified using the latest Read code or product code indicating smoking status prior to baseline (see S5 Table for codelists). Median height (calculated using height measurements prior to baseline and during follow-up) and the nearest weight measurement within 5 years prior to RA diagnosis were used to calculate body mass index (BMI) at baseline. Other disease groups for which influenza and pneumonia vaccinations are recommended were identified using Read codes: these included chronic respiratory disease, chronic heart disease, chronic kidney disease, chronic liver disease, chronic neurological disease, diabetes, asplenia or dysfunction of the spleen and other immunosuppression (see S6 Table for codelists). Patients were classified as meeting another clinical risk category at baseline if they had a Read code for at least one of these diseases prior to baseline. DMARD therapy was identified using product codes. The date of first DMARD prescription was identified for each patient, which may have been prior to the date of RA diagnosis. Using this information patients were classified by whether they were vaccinated prior to starting DMARD therapy, using data prior to RA diagnosis where necessary. The number of face-to-face GP consultations during the year prior to baseline was identified from the database, and the mean number of consultations was calculated.

### Analysis

For both vaccines, the number and percentage of patients who had at least one vaccination during follow-up, and who had their first vaccination prior to starting immunosuppressive therapy, stratified by age at baseline, was tabulated. The number of expected vaccinations was compared to the number of vaccinations received during follow-up, stratified by age at baseline. For the pneumococcal vaccination, this was described for the whole follow-up period. For the influenza vaccination only the first 5 years are reported due to the high number of vaccinations in some patients. The proportion of those vaccinated for each characteristic was calculated. Proportions vaccinated were compared by calculating the difference in the proportion vaccinated, and 95% confidence intervals (CI), between strata for each characteristic. The region of the practices was identified, and the percentage of patients who received at least one vaccination during follow-up was calculated for each practice, and displayed as a box and whisker plot by region.

The protocol for this study has been approved by Independent Scientific Advisory Committee for Medicines and Healthcare Regulatory Agency database research (Protocol number: 14_173). As this study used routinely collected anonymised electronic health records consent was not required.

## Results

**Fig 1:**
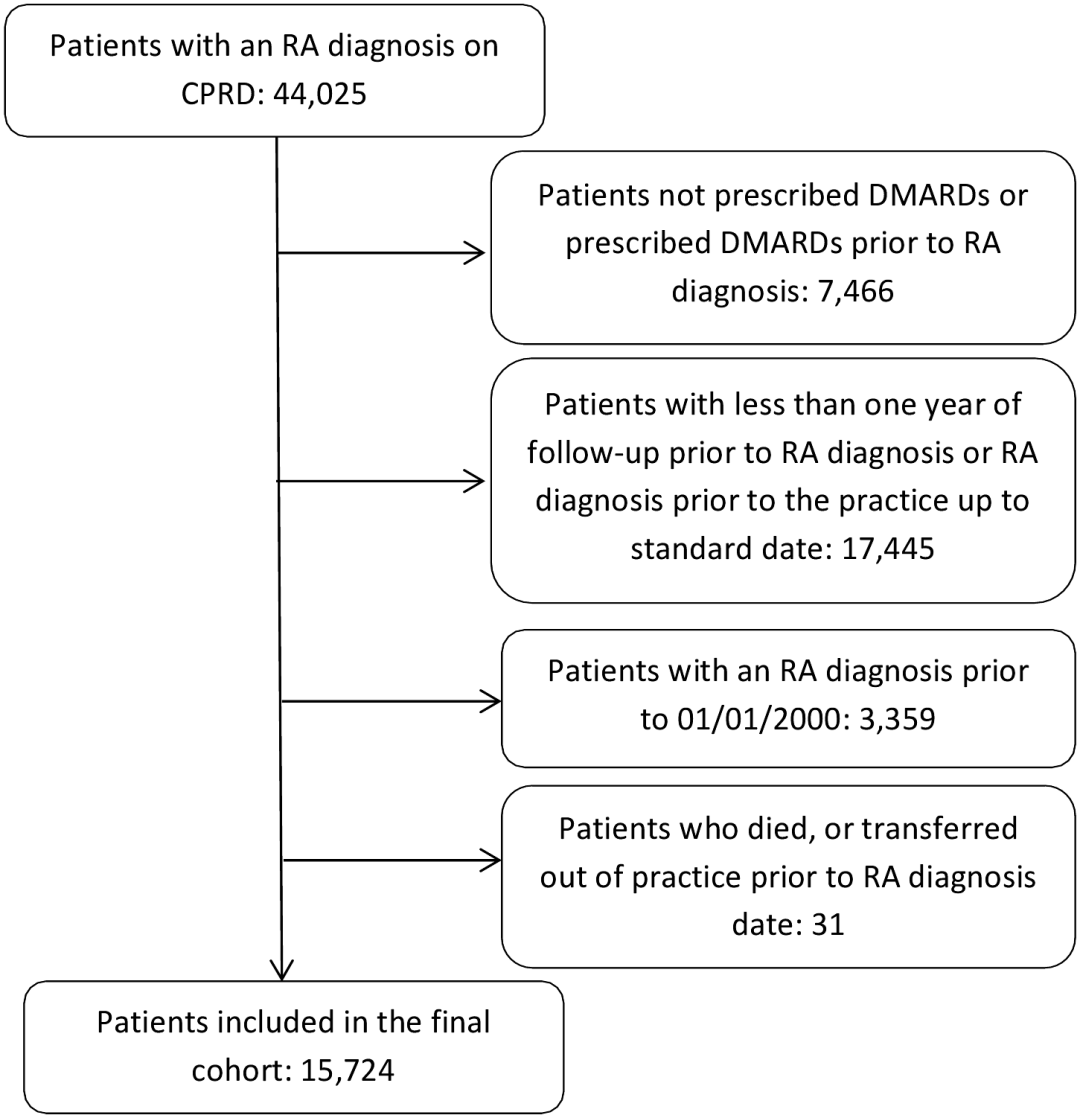
Flowchart of patients eligible for the cohort.

As shown in Fig 1 there were 15,724 patients with RA who met the criteria for inclusion in the study. These patients had a mean follow-up of 5.3 years (range: 0.003–14.0 years). Thirty-seven percent of the cohort were age 65 years and over at baseline, and 69% were female. Just over half (54%) of the cohort were smokers or ex-smokers at baseline, 20% had normal BMI, 26% met another clinical risk category at baseline and 22% visited their GP for a face-to-face consultation more than 5 times in the year prior to baseline. The DMARD most frequently prescribed during follow-up was methotrexate, and 50% of patients were prescribed oral glucocorticoids during follow-up (Table 1). 21% had a DMARD prescription prior to baseline, with patients prescribed DMARDs for a mean of 20.6 days (standard deviation: 17.7) prior to RA diagnosis. Overall, 12,492 (80%) patients had received at least one influenza vaccination during follow-up and 7,780 (50%) had received at least one pneumococcal vaccination during follow-up (Table 2).

**Table 1:** Characteristics of RA cohort, by vaccination status (N = 15724).

Characteristic	All	Vaccinated for influenza			Vaccinated for pneumonia		
	N (% of cohort)	N	Proportion vaccinated	Difference in proportion vaccinated (95% Confidence Interval)	N	Proportion vaccinated	Difference in proportion vaccinated (95% Confidence Interval)
Baseline age							
18–44 years	2910 (18.5)	1835	63.1	Reference	887	30.5	Reference
45–54 years	3005 (19.1)	2145	71.4	8.3 (5.9, 10.7)	1121	37.3	6.8 (4.4, 9.2)
55–64 years	4054 (25.8)	3302	81.5	18.4 (16.3, 20.5)	2270	56.0	25.5 (23.2, 27.8)
65–74 years	3502 (22.3)	3186	91.0	27.9 (25.9, 29.9)	2318	66.2	35.7 (33.4, 38.0)
Over 75 years	2253 (14.3)	2024	89.8	26.8 (24.6, 28.9)	1184	52.6	22.1 (19.4, 24.7)
Gender							
Female	10781 (68.6)	8525	79.1	Reference	5318	49.3	Reference
Male	4943 (31.4)	3967	80.3	1.2 (-0.2, 2.5)	2462	49.8	0.5 (-1.2, 2.2)
Baseline smoking							
Never smoker	6250 (39.8)	4919	78.7	Reference	3027	48.4	Reference
Ever smoker	8505 (54.1)	6877	80.9	2.2 (0.8, 3.4)	4293	50.5	2.0 (0.4, 3.7)
Missing	969 (6.2)	696	71.8	-6.9 (-9.9, -3.9)	460	47.5	-1.0 (-4.3, 2.4)
Baseline BMI							
Underweight	166 (1.1)	124	74.7	-5.3 (-12.1, 1.4)	69	41.6	-7.6 (-15.3, 0.1)
*Underweight (<18*.*5)*							
Normal	3102 (19.7)	2483	80.0	Reference	1526	49.2	Reference
*Normal (18*.*5–24*.*9)*							
Overweight	3502 (22.3)	2949	84.2	4.2 (2.3, 6.0)	1818	51.9	2.7 (0.3, 5.1)
*Overweight (25–29*.*9)*							
Obese	2607 (16.6)	2184	83.8	3.7 (1.7, 5.7)	1359	52.1	2.9 (0.3, 5.5)
*Obese (30–39*.*9)*							
Morbidly obese	372 (2.4)	297	79.8	-0.2 (-4.5, 4.1)	188	50.5	1.3 (-4.0, 6.7)
*Morbidly obese (> = 40)*							
Missing	5975 (38.0)	4455	74.6	-5.5 (-7.3, -3.7)	2820	47.2	-2.0 (-4.2, 0.2)
Met at least one other clinical risk category at baseline							
Yes	4034 (25.7)	3652	90.5	14.9 (16.1, 13.7)	2127	52.7	4.4 (6.2, 2.6)
No	11690 (74.3)	8840	75.6	Reference	5653	48.4	Reference
Number of face-to-face GP visits in year prior to baseline							
<5 visits	12329 (78.4)	9466	76.8	Reference	5916	48.0	Reference
5–9.9 visits	2684 (17.1)	2371	88.3	11.6 (10.1, 13.0)	1429	53.2	5.3 (3.2, 7.3)
10–14.9 visits	556 (3.5)	510	91.7	14.9 (12.5, 17.4)	335	60.3	12.3 (8.1, 16.4)
≥15 visits	155 (1.0)	145	93.5	16.7 (12.8, 20.7)	100	64.5	16.5 (8.9, 24.1)
Prescribed oral glucocorticoids during follow-up							
Yes	7792 (49.5)	6735	84.9	11.0 (12.3, 9.8)	4347	54.8	10.7 (12.3, 9.2)
No	7932 (50.5)	5757	73.9	Reference	3433	44.1	Reference
Prescribed methotrexate during follow-up							
Yes	11453 (72.8)	9517	83.1	13.4 (15.0, 11.9)	6006	52.4	10.9 (12.6, 9.2)
No	4271 (27.2)	2975	69.7	Reference	1774	41.5	Reference
Prescribed hydroxchloroquine during follow-up							
Yes	5593 (35.6)	4433	79.3	-0.2 (1.0, -1.6)	2749	49.2	-0.5 (1.1, -2.1)
No	10131 (64.4)	8059	79.5	Reference	5031	49.7	Reference
Prescribed sulfasalazine during follow-up							
Yes	7344 (46.7)	5869	79.9	0.9 (2.1, -0.4)	3773	51.4	3.6 (5.1, 2.0)
No	8380 (53.3)	6623	79.0	Reference	4007	47.8	Reference
Prescribed leflunomide during follow-up							
Yes	1838 (11.7)	1594	86.7	8.2 (9.9, 6.5)	1093	59.5	11.3 (13.7, 8.9)
No	13886 (88.3)	10898	78.5	Reference	6687	48.2	Reference
Prescribed other non-biologic DMARDs during follow-up							
Yes	1205 (7.7)	1047	86.9	8.1 (10.1, 6.0)	786	65.2	17.1 (19.9, 14.2)
No	14519 (92.3)	11445	78.8	Reference	6994	48.2	Reference

**Table 2:** Influenza and pneumonia vaccination uptake, and timing of vaccinations in relation to starting DMARD therapy (N = 15724).

	Influenza vaccination N (%)			Pneumonia vaccination N (%)		
	<65 years	≥65 years	Total	<65 years	≥65 years	Total
Ever had a vaccination						
Yes	7282 (73.0)	5210 (90.5)	12492 (79.5)	4278 (42.9)	3502 (60.9)	7780 (49.5)
No	2687 (27.0)	545 (9.5)	3232 (20.5)	5691 (57.1)	2253 (39.2)	7944 (50.5)
First vaccination prior to starting DMARDs^1^						
Yes	1415 (34.6)	2220 (74.2)	3635 (51.3)	1059 (24.7)	2199 (62.8)	3258 (41.9)
No	2677 (65.4)	771 (25.8)	3448 (48.7)	3219 (75.3)	1303 (37.2)	4522 (58.1)

^1^ For influenza vaccination: Includes patients whose first DMARD was during influenza season (September-March) as patients would not be vaccinated between April and August (N = 7083).

Of those aged below 65 years at baseline (N = 9,969), 7282 (73%) patients had received at least one influenza vaccination. Of those whose first DMARD was during the influenza season (N = 4,092) 1,415 (35%) were vaccinated prior to starting DMARD therapy. Of those expected to have up to 5 vaccinations (N = 4,309), 21%—31% received all expected vaccinations. There were 4,278 (43%) patients who received at least one pneumococcal vaccination, of whom 1,059 (25%) were vaccinated prior to starting DMARD therapy and 175 (1.8%) were revaccinated at least once (Tables 2 and 3).

**Table 3:** The expected versus received influenza and pneumonia vaccinations of the RA cohort, by age group.

Vaccinations expected	Vaccinations received	Influenza vaccination^1^ N (%)			Pneumonia vaccination^2^ N (%)		
		<65 years	≥65 years	Total	<65 years	≥65 years	Total
1	0/1	140 (68.6)	33 (24.3)	173 (50.9)	5691 (57.1)	2253 (39.2)	7944 (50.5)
	1/1	64 (31.4)	103 (75.7)	167 (49.1)	4103 (41.2)	3321 (57.7)	7424 (47.2)
	2+/1	-	-	-	175 (1.8)	181 (3.1)	356 (2.3)
2	0/2	470 (46.8)	106 (14.6)	576 (33.3)			
	1/2	263 (26.2)	133 (18.4)	396 (22.9)	-	-	-
	2/2	272 (27.1)	485 (67.0)	757 (43.8)			
3	0/3	395 (35.8)	92 (11.8)	487 (25.8)			
	1/3	195 (17.7)	65 (8.3)	260 (13.8)	-	-	-
	2/3	253 (22.9)	160 (20.4)	413 (21.9)			
	3/3	262 (23.7)	466 (59.5)	728 (38.6)			
4	0/4	297 (29.2)	60 (8.9)	357 (21.1)			
	1/4	133 (13.1)	28 (4.1)	161 (9.5)			
	2/4	128 (12.6)	47 (7.0)	175 (10.3)	-	-	-
	3/4	251 (24.7)	146 (21.6)	397 (23.5)			
	4/4	208 (20.5)	395 (58.4)	603 (35.6)			
5	0/5	269 (27.5)	62 (9.7)	331 (20.4)			
	1/5	108 (11.0)	20 (3.1)	128 (7.9)			
	2/5	93 (9.5)	21 (3.3)	114 (7.0)	-	-	-
	3/5	123 (12.6)	49 (7.6)	172 (10.6)			
	4/5	172 (17.6)	135 (21.0)	307 (19.0)			
	5/5	213 (21.8)	355 (55.3)	568 (35.1)			

^1^ Includes those who were expected to receive up to 5 vaccinations only (N = 7270).

^2^ Whole cohort (N = 15724)

Of those aged 65 years and above at baseline (N = 5,755), 5210 (91%) patients received at least one influenza vaccination. Of those whose first DMARD was during the influenza season (N = 2,991), 2,220 (74%) were vaccinated prior to starting DMARD therapy. Of those expected to have up to 5 vaccinations (N = 2,961), 55%-76% received all expected vaccinations. There were 3,502 (61%) patients who received at least one pneumococcal vaccination, of whom 2,199 (63%) received a vaccination prior to starting DMARD therapy and 181 (3.1%) were revaccinated at least once (Tables 2 and 3).

Of those aged 65 years and above at baseline (N = 5,755), 5210 (91%) patients received at least one influenza vaccination. Of those whose first DMARD was during the influenza season (N = 2,991), 2,220 (74%) were vaccinated prior to starting DMARD therapy. Of those expected to have up to 5 vaccinations (N = 2,961), 55%-76% received all expected vaccinations. There were 3,502 (61%) patients who received at least one pneumococcal vaccination, of whom 2,199 (63%) received a vaccination prior to starting DMARD therapy and 181 (3.1%) were revaccinated at least once (Tables 2 and 3).

The characteristics of the study cohort are shown in Table 1. Those who were vaccinated for influenza were older, 91% of those aged 65–74 years at baseline and 90% of those aged 75 years or older at baseline were vaccinated compared to 63% of those aged 18–44 years at baseline, giving differences of 28 percentage points (95% CI: 26, 30 percentage points) and 27 percentage points (95% CI: 25, 29 percentage points), respectively. This was similar for pneumococcal vaccinations, though those aged over 75 years at baseline had a lower percentage of coverage (53%) than those aged 65–74 years at baseline (66%). There were small differences in influenza vaccination coverage between baseline BMI categories, with higher coverage observed in the overweight and obese categories (84% in both categories) compared to the normal category (80%), a difference of only 4 percentage points. A similar pattern was observed for pneumococcal vaccination, though again the coverage was lower than for influenza vaccination. A greater proportion of those who met at least one other clinical risk category had been vaccinated, compared to those who did not meet another clinical risk category. This was true for both influenza and pneumococcal vaccinations, though there was a greater difference for those vaccinated for influenza, with a difference 15 percentage points (95% CI: 16, 14 percentage points) compared to a difference of 4 percentage points (95% CI 6, 3 percentage points) for pneumococcal vaccinations. Those who visited their GP more often were more likely to be vaccinated with either vaccine. A greater proportion of those prescribed oral glucocorticoids, methotrexate, leflunomide and other DMARDs were vaccinated, for both types of vaccine. A greater proportion of those prescribed sulfasalazine had a pneumococcal vaccination, though this was not true for influenza vaccination. There were no differences in the proportion vaccinated in those prescribed hydroxychloroquine. There were small differences in the proportion vaccinated by smoking status. Ever smokers were vaccinated with either vaccine slightly more than never smokers. The proportion vaccinated with either vaccine did not differ by gender.

**Fig 2:**
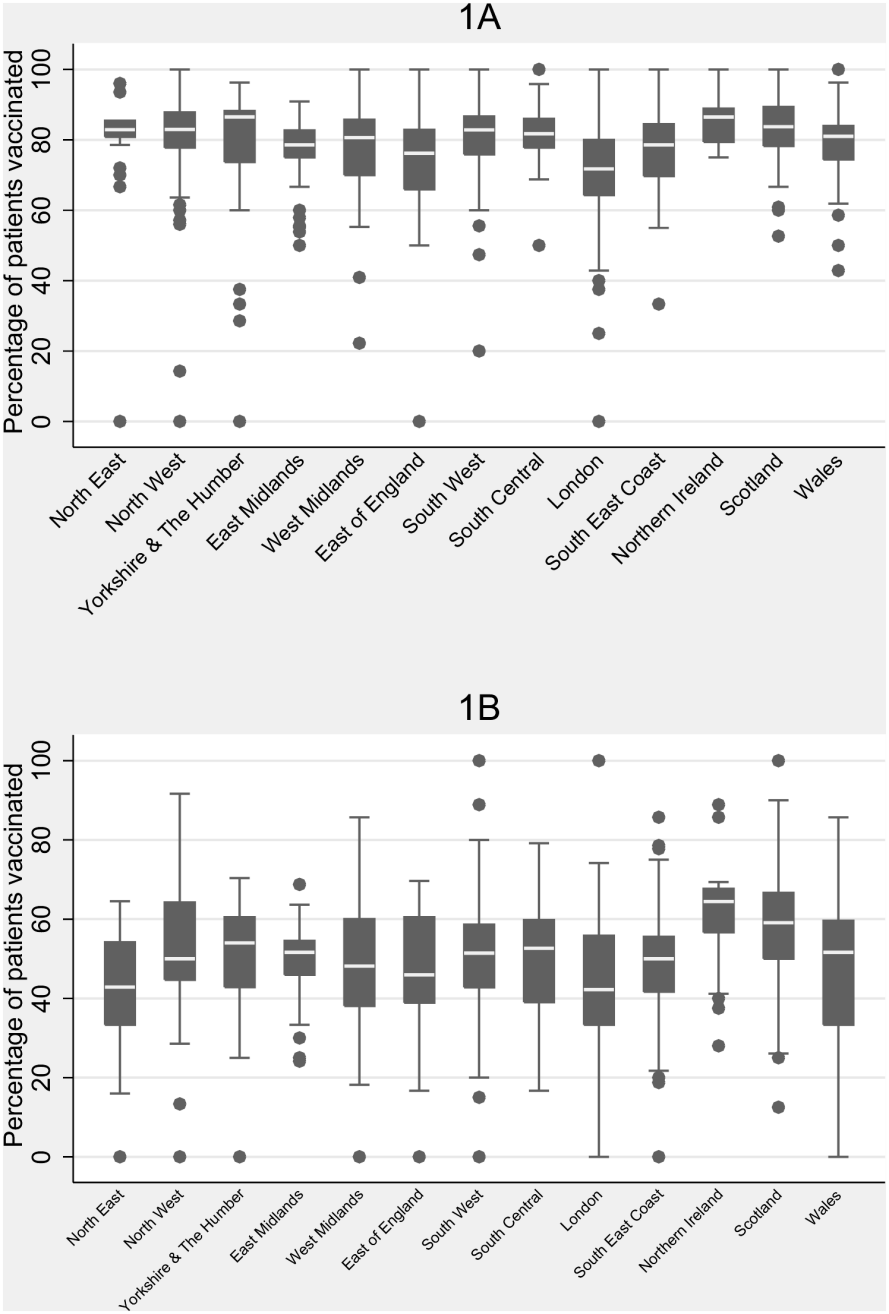
Box and whisker plots of influenza and pneumococcal vaccination uptake by region. Box and whisker plots showing the percentage of patients within a practice receiving at least one influenza vaccination (1A) or pneumonia vaccination (1B), by region. Box plots represent the median (central line), interquartile range (box), range, excluding outliers (whiskers) and outliers (dots) of the percentage of patients within a practice who receive at least one vaccination during follow-up.

Regional differences were observed in both influenza and pneumococcal vaccination coverage. The highest coverage of influenza and pneumococcal vaccination was in Northern Ireland (86% and 61% respectively). The lowest coverage was in London (72% and 43% respectively) (Fig 2). The differences in regional variation were unchanged when stratified by age.

## Discussion

This large cohort study, using electronic health records from GPs in the UK, has shown that one in five immunosuppressed patients with RA did not receive any influenza vaccinations during follow-up, and up to two thirds were not vaccinated annually. Half of those vaccinated received their first vaccination prior to starting DMARDs. Uptake of pneumococcal vaccinations was much lower, less than half were vaccinated, and less than 3% of patients received booster vaccinations. Of those vaccinated, 50% were vaccinated prior to starting DMARDs. Those who were younger, who did not meet another clinical risk category, and who visited their GP less often were least likely to be vaccinated. There was some variation in the proportion who received at least one vaccination by region with Northern Ireland having the highest coverage, and London having the lowest coverage.

The EULAR recommendations for vaccination in patients with rheumatic diseases [5], and the UK vaccination guidelines for patients who are immunosuppressed [6, 24], recommend an annual influenza vaccination and a pneumococcal vaccination. To our knowledge, this is the first large study to describe the uptake of influenza and pneumococcal vaccinations, and timing of vaccinations in relation to starting DMARD therapy, in patients with incident RA in the UK. There have been a small number of single centre audits of vaccination uptake in patients with rheumatic diseases in the UK [9–13, 19, 20] (Table 4), only 3 of these were investigating patients with RA specifically [10–12]. The audits had between 64 and 169 patients and found influenza vaccination uptake to be between 56% and 79% [9–13, 19, 20]. Pneumococcal vaccination rates were lower at between 33% and 43% [11, 13, 20]. Though in one study influenza vaccination uptake varied between 54% and 93%, and pneumococcal vaccination varied between 38% and 64% depending on the type of DMARD the patient was taking and whether they had any other risk factors for vaccination [9]. Another study used data from a cohort study, which included 43 UK patients, and described vaccination uptake [16]. The study found that 84% of UK patients had ever received an influenza vaccination, only 30% had optimal use, and 44% of UK patients had ever received a pneumococcal vaccination. These results were similar to this study. The Department of Health estimates that 52% of those on immunosuppressive medication aged 18–64 years, were vaccinated for influenza in 2013/14 and 73% of those aged 65 years and over were vaccinated for influenza [21]. This study has found slightly higher numbers had received at least one influenza vaccination; compared to previous audits, however when broken down by the number of expected and received vaccinations the patients receiving all vaccinations expected was lower than the previous audits at between 33%-50% for those with 5 years follow-up, which is suboptimal. The percentage of patients who received a pneumococcal vaccination during follow-up was similar to previous audits.

**Table 4:** Summary of studies of influenza and pneumococcus vaccination uptake in the UK.

Author	Type of study	N	Disease group	Influenza vaccine uptake	Pneumococcal vaccine uptake
Pradeep et al (2006)	Audit	64	Rheumatoid arthritis	63%	43%
Doe et al (2007)	Audit	169	Rheumatic diseases	79%	34%
Thomas et al (2004)	Audit	111	Rheumatic diseases	70%	33%
Bridges et al (2003)	Audit	129	Rheumatoid arthritis	56% (of those taking MTX (n = 59))	-
Clarke et al (2011)	Audit	71	Rheumatoid arthritis	~70%	-
Saravana et al (2004)	Audit	100	Rheumatic diseases	77%	-
Sowden et al (2007)	Audit	101	Rheumatic diseases	54%-93%	38%-64%
Hmamouchi et al (2015)	Cohort	43 (UK patients)	Rheumatoid arthritis	84%	44%

In previous studies patients were only expected to receive one pneumococcal vaccination, with no boosters. Although there are guidelines recommending boosters every 5 years [7], the green book for GPs in the UK recommend only vaccinating once [6], and the EULAR guidelines state that it is unknown whether boosters are required [5], so this may explain why patients have not received booster vaccinations and perhaps needs clarification.

This study also found that those with another indication for vaccination, in particular being aged 65 years or over had higher rates of vaccination, which was similar to previous audits. Only one previous study in the US had reported the proportion of patients with rheumatic diseases who received a pneumococcal vaccination prior to starting immunosuppressive therapy [15]. They found 37% of patients were vaccinated prior to starting immunosuppressive therapy, which was similar to this study where 42% of those vaccinated received a pneumococcal vaccination prior to starting DMARD therapy. There was wide variation by age, those below 65 years of age were much less likely to have been vaccinated prior to starting DMARDs (only 35% and 25% for influenza and pneumonia vaccination, respectively, for those below 65 years of age compared to 74% and 63% in those over 65 years of age). Future research is required to see whether this confers a clinical benefit in terms of reducing the incidence of infection. Because DMARDs can be initiated in hospitals with GP prescribing only after the first few months’ hospital treatment, the proportions of patients vaccinated prior to DMARD therapy may be an over-estimate. It may be more difficult to vaccinate for influenza prior to starting DMARDs, as vaccination takes place at a specific point in the year, however pneumonia vaccination can take place all year round, so should be easier to accomplish.

Interestingly there was some variation in uptake of vaccinations by region and the trend was similar to the variation seen in the NHS immunisation statistics for England 2013/14 [25]. Northern Ireland had the highest rates of vaccination and London had the lowest rates of vaccination, perhaps due to regional differences in the promotion of vaccination.

In the UK there are incentives for GPs to provide influenza vaccinations for those aged 65 years and over and those with coronary heart disease, a history of stroke or transient ischaemic attacks, diabetes and chronic obstructive pulmonary disease, through Quality and Outcomes Framework (QOF) targets. Pneumonia vaccinations for those aged 65 years and over, and influenza vaccinations for those at-risk but not on the QOF indicators (including those who are immunosuppressed), are implemented through enhanced services. These provide GPs with payment for immunisations, though do not specify targets for how many should be vaccinated. This may explain the low vaccination rates observed, particularly for pneumonia vaccinations, where the enhanced services only covers those aged 65 years or over and it does not cover at risk groups. The UK influenza and pneumococcal vaccination booklets are not specific regarding RA, and the decision on whether to vaccinate individual immunosuppressed patients is left to clinician discretion. Therefore, it may be beneficial for rheumatologists to provide more input into the vaccination process. For example, they may wish to consider administering vaccines themselves prior to initiating immunosuppressive therapy, or provide GPs with clear advice on when vaccines should be administered. Regardless, experience tells us it is essential that both approaches should be implemented and resourced or they will be ineffective.

The study has several advantages—it used a large sample of patients with RA therefore the study population is likely to be representative of patients with RA in the UK. The data used came from electronic medical records which were recorded at the time of the visit so there should not be inconsistencies in the way GPs recorded the data or in how patients reported their symptoms. The data is “real-world data” and contains information on administered vaccinations, rather than being self-reported, therefore is likely to be accurate and free from recall bias. In the UK vaccinations primarily take place in primary care therefore most vaccinations will have been identified using primary care electronic medical records. There are however some limitations to be considered when interpreting the results. There may be some misclassification with respect to the identification of diseases such as RA, within CPRD as these are coded by the practices. However, vaccinations should be coded accurately as the product codes are generally only added to the health records after administration by a clinician, hence representing administration and not prescription. In addition, the vaccination codes are used to identify QOF and enhanced services compliance to determine payment, so GPs have an incentive to ensure they are accurate. Studies have shown that patients with RA on biologic DMARD therapy were more likely to have received a pneumococcal vaccination [8, 16]. Biologic DMARD therapy is not captured on the CPRD database as these are prescribed in secondary care, therefore we do not know what influence this had on vaccination uptake. There was some missing data for BMI and smoking as this is collected opportunistically, however the amount of missing data was small, particularly for smoking.

In conclusion, despite international recommendations, this study has found that many patients with RA in the UK are not being immunised regularly for influenza, and often not at all for pneumonia. Many patients are not being immunised prior to starting DMARD therapy. The patients most often being missed are those who are below 65 years of age and who do not have another disease for which vaccination is recommended.

## Supporting Information

S1 Table Rheumatoid arthritis codelist. (XLSX)

S2 Table Disease-Modifying Anti-Rheumatic Drug codelist. (XLSX)

S3 Table Influenza vaccination codelists. (XLSX)

S4 Table Pneumococcal vaccination codelists. (XLSX)

S5 Table Smoking codelists. (XLSX)

S6 Table Clinical risk group codelists. (XLSX)
